# Keeping Up Standards: An Audit of Adherence to Admission Standards on Acute Mental Health Wards in NHS Lanarkshire

**DOI:** 10.1192/bjo.2024.557

**Published:** 2024-08-01

**Authors:** Louisa Adam, Shaina Dillon, Emma Docherty, Francesca Guarino, Leanne Lindsay

**Affiliations:** 1 University Hospital Wishaw, NHS Lanarkshire, United Kingdom; 2 University Hospital Hairmyres, NHS Lanarkshire, United Kingdom; 3 Woodland View, NHS Ayrshire and Arran, United Kingdom; 4 NHS Education for Scotland, NHS Lanarkshire, United Kingdom

## Abstract

**Aims:**

We audited the adherence to part of the minimum admission standards for Mental Health, Learning Disabilities and Addictions Services (MHLDA) for 6 acute wards, across two sites (UHH and UHW) in NHS Lanarkshire. We focussed on the section of the standards that the admitting junior doctor/ANP is responsible for. This comprised:

- An admission assessment (including presenting complaint, history of current episode of illness, medication, mental state examination and risk assessment).
- Physical health assessment (examination, bloods, ECG, VTE assessment), medicine reconciliation and prescribing on HEPMA - within 12 hours.

**Methods:**

Five individuals collected data across both sites and both cycles. For our first cycle, all admissions in March 2023 were retrospectively reviewed, a total of 94 admissions (UHH 47, UHW 47). Electronic notes/systems were reviewed (Morse, Clinical Portal, Hepma, Trakcare).

This first cycle demonstrated poor adherence to the minimum admissions standards. A proforma for admission statement was created, including prompts for the admission assessment and for the components of the physical health assessment, medicines reconciliation and prescribing. Presentations were made at postgraduate teaching and at ANP teaching. The majority of people were unaware of the existence of the admission standards or did not know where to find them. The admission standards document and the proforma were circulated via email and added to the shared R drive. A second cycle was completed, reviewing all admissions in July 2023, a total of 74 admissions (UHH 41, UHW 33). The proforma has now been included in the induction material for new doctors.

**Results:**

Following interventions, there was improvement in completion of admission statement (90% vs 81%). There was improvement in the inclusion of all components, most notably MSE (91% vs 71%) and risk assessment (59% vs 18%). Where the proforma was used (57%), all aspects of admission statement were present (97–100%). When not used, there was variable inclusion of the different components (7–90%). There was improvement in the completion of all components of physical health assessment (except small decrease in medicine reconciliation). In every case of missing components with no documentation as to why, the proforma had not been used.

**Conclusion:**

Development of a proforma for admission assessment has led to improved completion of admission assessment, physical health assessment, medicines reconciliation and prescribing within 12 hours. Qualitative feedback is being sought on the proforma from junior doctors, ANPs and senior medics to guide next steps and further improvements. Review of the admissions standards guidance is now due.

